# Association of triglyceride–glucose and obesity-derived indices with the risk of aortic stenosis among individuals in cardiovascular–kidney–metabolic syndrome stages 0–3: a prospective cohort study from the UK Biobank

**DOI:** 10.3389/fendo.2026.1844215

**Published:** 2026-07-07

**Authors:** Jiacheng Ding, Xinyu Cai, Jingqian Li, Yiyin Gao

**Affiliations:** 1 The Second Hospital of Jilin University, Changchun, Jilin, China; 2 Jilin University, Changchun, Jilin, China

**Keywords:** aortic stenosis, cardiovascular-kidney-metabolic, obesity, triglyceride, UK Biobank

## Abstract

**Background:**

Cardiovascular-kidney-metabolic (CKM) syndrome emphasizes the interplay between metabolic dysfunction, kidney injury, and cardiovascular abnormalities. The triglyceride-glucose (TyG) and its obesity-related derivatives have emerged as simple biomarkers for insulin resistance, yet their association with aortic stenosis (AS) risk in CKM syndrome stages 0–3 remains unclear.

**Methods:**

This prospective cohort study included 363,907 participants with CKM stages 0–3 from the UK Biobank. We calculated six TyG-related indices (TyG, TyG-BMI, TyG-Waist, TyG-WHR, TyG-WHtR, and TyG-ABSI) and evaluated their associations with incident AS using Cox proportional hazards and competing risk models. Mediation analysis assessed the role of systolic blood pressure (SBP), and incremental predictive value was evaluated using C-index and net reclassification index (NRI).

**Results:**

During 15.25 years of follow-up, 3,326 AS cases occurred. After full adjustment, each 1-SD increment in TyG-related indices was associated with 8-24% increased AS risk. Compared with the lowest tertile, the highest tertile showed 1.13-1.77-fold increased risk. All indices demonstrated linear dose-response relationships with AS. SBP mediated 12.1-18.1% of these associations. TyG-WHtR showed the greatest predictive improvement (C-index: 0.806; NRI: 0.308).

**Conclusions:**

TyG-related indices, particularly TyG-WHtR, are independently associated with AS risk in CKM syndrome stages 0-3, offering potential for early risk stratification and targeted prevention strategies.

## Introduction

By 2021, the global occurrence of cardiovascular disease (CVD) had risen to 523 million, substantially contributing to worldwide mortality and the escalating burden of healthcare expenditures. Accumulating evidence has demonstrated strong associations between CVD, metabolic diseases (MetS), as well as chronic kidney disease (CKD) (1, 2). To further characterize the complex interplay among these diseases, cardiovascular–kidney–metabolic (CKM) syndrome was introduced. This report emphasized that CKM, as a systemic condition, significantly increased the risk of cardiovascular complications and dysfunction across multiple organ systems (2). The CKM framework underscores the synergistic interactions among metabolic dysregulation, renal impairment, and cardiovascular abnormalities, supporting a unified approach to disease prevention and positioning CKM as a major research focus (2). CKM syndrome is categorized into 5 stages, with 0–3 considered the preclinical phase (>90%) (3), making early screening and intervention critical for preventing cardiovascular and renal diseases.

Insulin resistance is a metabolic disturbance characterized by impaired insulin-mediated glucose-lowering effects in peripheral tissues. Extensive research has established insulin resistance as a standalone risk factor for CVD (4). For example, a study of 5,301 participants demonstrated that elevated insulin resistance was connected to more than a 50% greater CVD risk (4). Similarly, a prospective study among Chinese population showed that CVD risk rose proportionally with an accumulated metabolic score of insulin resistance (5). Moreover, a Mendelian randomization analysis confirmed that genetically proxied insulin resistance increased the risk of hypertension, a well-recognized traditional CVD risk factor (6).

Although hyperinsulinemic–euglycemic clamp (HIEC) is regarded as the definitive benchmark for quantifying insulin resistance. But its high cost, technical complexity, and need for specialized personnel limit its feasibility in routine clinical practice (7). In recent years, simpler clinical indices—such as the homeostatic model assessment of insulin resistance (HOMA-IR) and the quantitative insulin sensitivity check index (QUICKI)—have been validated as robust proxy indicators (8). Moreover, the triglyceride–glucose (TyG) index has gained considerable attention as a simple and accessible biomarker of insulin resistance. Numerous studies have reported significant associations of TyG and CVD risk. For instance, a prospective study suggested that elevated TyG levels were strongly correlated with elevated risks of myocardial infarction and stroke (9).

Meanwhile, obesity has become a prominent issue in global public health. A 2022 report noted that more than one billion individuals worldwide were living with obesity (10). Specifically, multiple studies have shown that obesity not only correlates with established CVD risk factors, but also directly contributes to CVD development (11, 12). Findings from a cohort with 26-year follow-up indicated that obesity independently increased the risk of CVD (13). Consequently, TyG–obesity composite indices have attracted growing research interest. Several studies have shown that TyG–obesity indices outperform the TyG alone on estimating CVD risk (14, 15). Collectively, previous investigations suggest substantial progress in the utility of TyG and its obesity-related derivatives for cardiovascular risk prediction.

However, the majority of prior investigations have centered on either broad populations or cohorts defined by specific diseases, and only a limited number have examined linkage on specific cohorts (16). Although they provide meaningful insights for primary CVD prevention in early CKM populations, evidence remains lacking for specific cardiovascular outcomes. In recent years, the prevalence of degenerative valvular heart disease (VHD) has risen substantially with aging populations. Aortic stenosis (AS), the most common manifestation of VHD, is characterized by restricted leaflet opening due to structural abnormalities, leading to impaired ventricular outflow and significant hemodynamic burden (17). A prior research has demonstrated that AS severity strongly correlates with elevated mortality risk (18), with aortic valve disease resulted in 127,000 deaths worldwide estimated by a global burden study in 2019 (19).

Notably, no effective pharmacologic therapies or preventive strategies for AS currently exist in clinical practice (20). Although aortic valve replacement (AVR) can substantially improve clinical outcomes in patients with severe AS, more than 40% of affected individuals do not undergo AVR—either owing to late diagnosis, lack of symptoms, or ineligibility for surgery (20, 21)—resulting in missed opportunities for timely intervention (22). Thus, timely recognition of patients at elevated risk and deployment of targeted prevention measures are of critical clinical relevance. Although age, smoking, hypertension, and lipoprotein(a) have been linked to the development of AS, their predictive accuracy for incident AS remains suboptimal (23, 24). Therefore, exploring whether TyG and its obesity-derived index can facilitate early diagnosis and risk stratification for AS in CKM stage 0–3 populations is of considerable importance.

In this context, a large-scale prospective study was performed on UK Biobank to comprehensively figure out the linkage of TyG and its obesity-derived index with AS risk among CKM stages 0–3 patients. We further examined the possible mediating function of systolic blood pressure (SBP) and assessed whether incorporating these indices improved the predictive performance of traditional AS risk models. Our findings offer evidence on early AS risk identification and offer theoretical support for precision prevention among specific population.

## Methods

### Study population

This study was carried out using data from UK Biobank (UKB), an extensive prospective cohort that recruited over 500,000 individuals aged 40–69 years from 22 centers including England, Scotland, and Wales between 2006 and 2010. Participants have been followed continuously, with extensive data collected via touchscreen surveys, clinical examinations, and analysis of biological specimens. Details of UKB’s study design and data collection procedures are available online. The study was approved by the National Health Service National Research Ethics Service (ref: 11/NW/0382) and the Institutional Review Board of Tulane University (study number: 2018-1872). Written informed consent was obtained from all patients. Additional methodological details have been published previously (25, 26). The project ID for the present study was 629227.

408,984 individuals with fully available CKM data were first enrolled. We subsequently excluded individuals who met any of the following criteria: (i) CKM stage 4 (n = 36,959); (ii) prior diagnosis of rheumatic valvular disease (n = 59); (iii) prior diagnosis of non-rheumatic valvular disease (n = 3,430); (iv) congenital valvular disease or Marfan syndrome (n = 32); (v) endocarditis with valvular involvement; (vi) pulmonary valve disease; (vii) missing data for TyG or TyG–obesity indices (n = 1,882); (viii) missing covariates (n = 16,770). Ultimately, 363,907 patients were incorporated into analysis (**Supplementary Figure 1**). All diagnoses were ascertained using ICD-10 codes (**Supplementary Table 1**).

### Calculation of TyG-related indices

Peripheral blood specimens were obtained to measure biochemical indexes, including glucose, triglycerides (TG), and high-density lipoprotein cholesterol (HDL-C). The samples were processed by fractionating them and storing each component at −80 °C. All assessments were conducted on the Beckman Coulter AU5800 automated clinical chemistry analyzer. Detailed methods for specimen collection, handling, and quality assurance have been comprehensively described previously. The calculation formulas are:

$\text{TyG}=\ln\frac{TG(\frac{mg}{dL})\times Glucose(\frac{mg}{dL})}{2}$;

$\text{TyG}-\text{BMI}=\text{TyG}\times \text{BMI}=\text{TyG}\times \frac{Weight(kg)}{Height{\left(m\right)}^{2}}$;

$TyG-\text{WHR}=\text{TyG}\times \text{WHR}=\text{TyG}\times \frac{Waist\;circumference(cm)}{Hip\;circumference\;(cm)}$;

$TyG-\text{WHtR}=\text{TyG}\times \text{WHtR}=\text{TyG}\times \frac{Waist\;circumference(cm)}{Height(cm)}$;


$$TyG-\text{ABSI}=\text{TyG}\times \text{ABSI}=\text{TyG}\times \frac{Waist\;circumference(cm)}{BMI{\left(\frac{kg}{m^{2}}\right)}^{2/3}\times Height{\left(m\right)}^{1/2}}$$

### Definition of CKM syndrome

CKM syndrome highlights the interactive effects of metabolic and renal impairment, as well as cardiovascular abnormalities and is grouped into 5 stages (0–4).

Stage 0: Participants free of CKM risk factors, including body mass index (BMI), waist circumference, blood glucose, systolic and diastolic blood pressure, lipid profile, and with no CKD or CVD.

Stage 1: Presence of any of the following: (1) BMI ≥23 kg/m² for Asian participants or ≥25 kg/m² for other ethnicities; (2) waist circumference ≥80 cm for Asian females, ≥90 cm for Asian males; ≥88 cm for females and ≥102 cm for males of other ethnicities; (3) prediabetes: HbA1c 5.7–6.5% or fasting glucose 100–126 mg/dL.

Stage 2: Individuals with moderate-to-high-risk CKD, characterized as eGFR 30–60 mL/min/1.73 m², along with confirmed metabolic risk factors including hypertension, diabetes, TG ≥135 mg/dL, and metabolic syndrome. Metabolic syndrome was characterized by fulfilling more than three of these criteria: increased waist circumference; HDL-C <40 mg/dL in males or <50 mg/dL in females; TG >150 mg/dL; systolic BP ≥130 mmHg, diastolic BP ≥80 mmHg, or antihypertensive treatment; or prediabetes.

Stage 3: Individuals with very high-risk CKD (eGFR <30 mL/min/1.73 m²) or those with a 10-year CVD risk >20% according to the PREVENT risk model (16, 27).

Stage 4: Individuals with overlap of stages 0–3 who had established CVD, including heart failure, atrial fibrillation, coronary heart disease, stroke, or peripheral arterial disease. Detailed CKM classification is provided in **Supplementary Table 2**.

### Outcome definition

The main outcome of this study was incident AS among CKM stages 0–3 participants. AS events were ascertained using hospital diagnosis records linked to ICD-10 codes I35.0 and I35.2, as listed in **Supplementary Table 1**. Participants were followed until the occurrence of AS, death, loss to follow-up, or the end of hospital episode statistics, with the first event being considered. Mortality data were retrieved from death certificates maintained by NHS Information Centre (England and Wales) and the Scottish NHS Central Register (Scotland). Hospital admission was linked to Health Episode Statistics (England and Wales) and Scottish Morbidity Record (SMR01) database (Scotland). Follow-up for disease diagnoses was completed on July 1, 2024, and mortality data were updated through July 8, 2024.

### Covariates

Covariates included sociodemographic characteristics, lifestyle factors, biochemical markers, and comorbidities. Demographics and lifestyle factors, including age, sex, ethnicity, and smoking status, were obtained through touchscreen questionnaires and baseline interviews. Systolic blood pressure (SBP) was measured by trained personnel, with the maximum of multiple readings used. Lipoprotein(a) was measured from baseline peripheral blood samples. Histories of diabetes, emphysema, and hyperlipidemia were ascertained via self-report and linked ICD-10-coded health records, including hospitalizations, death records, primary care data, and UK Biobank self-reports. Medication use (lipid-lowering agents, antihypertensives, and insulin) was obtained via interview. eGFR was obtained by 2021 CKD-EPI equation without race adjustment (**Supplementary Table 3**). Urinary albumin-to-creatinine ratio (UACR) was calculated as the ratio of urinary albumin concentration (mg/L) to urinary creatinine concentration (g/L), expressed in mg/g.

Race was categorized as White, Black, Asian, or other ethnicities. Smoking status was classified as never, ever, or current. BMI was categorized as low (18.5–25.0 kg/m²), medium (25.0–30.0 kg/m²), or high (≥30.0 kg/m²). Waist circumference was categorized as low (<94 cm for males; <80 cm for females), medium (94–101.9 cm for males; 80–87.9 cm for females), or high (>102 cm for males; >88 cm for females). Waist-to-height ratio (WHtR) was categorized as low (<0.5), medium (0.5–0.6), or high (>0.6) (24). Waist-to-hip ratio (WHR) and a body shape index (ABSI) were divided into low (lowest quintile), medium (quintiles 2–3), and high (highest quintile). High-sensitivity C-reactive protein (hsCRP) was measured from baseline peripheral blood samples as a marker of systemic chronic inflammation. Physical activity level was quantified as the sum of moderate and vigorous physical activity in metabolic equivalent of task (MET) minutes per week, derived from the International Physical Activity Questionnaire (IPAQ) short form administered at baseline. In UK Biobank, the dietary scoring criteria were modified according to the AHA recommendations to adapt to the data availability in the UK Biobank (28). Participants’ dietary intake patterns were collected through a touchscreen questionnaire. These patterns included higher intake of fruits, vegetables, whole grains, fish, and chicken, as well as reduced intake of processed meat, unprocessed meat, cheese, and butter. We derived the dietary scores on the basis of the dietary frequency items collected from the questionnaire and the HEI-2015 quintile method.

### Statistical analysis

Baseline characteristics of CKM stages 0–3 participants were summarized according to AS status. Continuous variables were presented as mean ± SD, whereas categorical variables were reported as counts and percentages. Between-group differences were examined by t-tests, Kruskal–Wallis rank-sum tests and Pearson’s chi-square tests. Cumulative incidence of AS was estimated using Kaplan-Meier curves, along with log-rank tests contrasting differences across tertiles of TyG and obesity-derived indices.

Cox regression and Fine-Gray competing risk models were utilized to examine correlations of TyG-related indices with incident AS among CKM 0–3 participants. Model 1 adjusted for age, sex, and ethnicity, whereas Model 2 additionally adjusted for SBP, eGFR, lipoprotein(a), smoking status, hyperlipidemia, history of diabetes, and emphysema. In competing risk models, all-cause mortality was considered as a competing event. Hazard ratios (HRs) and 95% confidence intervals (CIs) were estimated per SD increase of continuous TyG-related indices and across tertiles, using the lowest tertile as reference. Restricted cubic spline (RCS) functions nested within Cox models were applied to examine dose–response relationships between six TyG-related indices and AS risk.

To shed light on the mediating effect of SBP on the connection between TyG-related indices and AS, multivariable linear regression models assessed the relationship between a 1-unit increase in each TyG index and SBP, adjusting for the same covariates as Model 2. Mediation analysis then estimated the proportion of AS risk mediated by SBP. Statistical significance of the mediation effect was evaluated using bootstrap resampling (500 iterations) to estimate 95% CIs.

Two predictive models for AS were developed: a baseline model including the same variables as Model 2, and an extended model incorporating each TyG-related index separately. Model performance improvement was evaluated using Harrell’s C-index and the net reclassification index (NRI).

Subgroup and sensitivity analyses were completed to ensure robustness, which were stratified by age (<60 vs. ≥60 years), sex, ethnicity (White vs. non-White), CKM stage (0–3), smoking (never, ever, current), BMI (<30 vs. ≥30 kg/m²), diabetes, hypertension, and eGFR. Sensitivity analyses included exclusion of patients undergo AS events within the first 2 years and further adjustment for medication use (antihypertensives, lipid-lowering agents, and insulin). To further address potential residual confounding effects, we conducted additional sensitivity analyses in the subset of participants who had the corresponding data. In these analyses, we additionally and separately adjusted for one of the following variables on the basis of all covariates included in Model 2: UACR, high-sensitivity C-reactive protein (hsCRP), moderate-to-high physical activity level, and dietary quality.

## Results

### Baseline characteristics

A total of 363,907 CKM stages 0–3 patients were included, comprising 162,951 males and 200,956 females. Baseline features are demonstrated in **Table 1**. Compared to individuals without incident AS during follow-up, those who developed AS were older, predominantly White, male, and more likely to be former or current smokers. They also exhibited lower eGFR, higher SBP and DBP, as well as elevated BMI, waist circumference, hip circumference, WHR, ABSI, WHtR, TyG, and its obesity-derived indices. Individuals with incident AS were more possibly to be classified as CKM stages 2 or 3 and had higher prevalence of diabetes, hypertension, hyperlipidemia, bronchitis, asthma, emphysema, greater use of lipid-lowering, antihypertensive, and antidiabetic medications.

**Table 1:** Baseline characteristics of the study participants.

Characteristic	Overall (n=363,907)	Non-AS (n=360,581)	AS (n=3326)	P-values
**Age, y**	56.14 ± 8.09	56.08 ± 8.08	62.28 ± 5.54	<0.001
**Male**	162,951 (44.8)	160,913 (44.6)	2038 (61.3)	<0.001
**Race**				<0.001
White	343,984 (94.5)	340,729 (94.5)	3255 (97.9)	
Asian	8254 (2.3)	8211 (2.3)	43 (1.3)	
Black or Black British	6040 (1.7)	6027 (1.7)	13 (0.4)	
Other ethnic	5629 (1.5)	5614 (1.6)	15 (0.5)	
**Smoke Status**				<0.001
Never smoker	203,070 (55.8)	201,649 (55.9)	1421 (42.7)	
Ever smoker	123,089 (33.8)	121,588 (33.7)	1501 (45.1)	
Current smoker	37,748 (10.4)	37,344 (10.4)	404 (12.1)	
**Height, cm**	168.51 ± 9.28	168.51 ± 9.28	169.11 ± 8.98	<0.001
**BMI kg/m^2^**	27.23 ± 4.69	27.21 ± 4.68	29.44 ± 5.37	<0.001
**BMI >=30.0kg/m^2^**	83,870 (23.0)	82,568 (22.9)	1302 (39.1)	<0.001
**Waist, cm**	89.73 ± 13.24	89.66 ± 13.22	97.43 ± 14.00	<0.001
**Hip, cm**	103.13 ± 9.06	103.11 ± 9.04	106.08 ± 10.40	<0.001
**WHR**	0.87 ± 0.09	0.87 ± 0.09	0.92 ± 0.09	<0.001
**ABSI**	7.65 ± 0.54	7.65 ± 0.54	7.88 ± 0.52	<0.001
**WHtR**	0.53 ± 0.07	0.53 ± 0.07	0.58 ± 0.08	<0.001
**eGFR mL/min/1.73 m2**	95.63 ± 14.08	95.71 ± 14.03	87.40 ± 16.10	<0.001
**SBP mmHg**	141.45 ± 19.44	141.36 ± 19.40	151.62 ± 20.14	<0.001
**DBP mmHg**	84.31 ± 10.44	84.30 ± 10.44	85.30 ± 10.94	<0.001
**Diabetes**	15,548 (4.3)	15,060 (4.2)	488 (14.7)	<0.001
**Hypertension**	91,338 (25.1)	89,650 (24.9)	1688 (50.8)	<0.001
**Hyperlipidemia**	44,905 (12.3)	43,947 (12.2)	958 (28.8)	<0.001
**Bronchitis**	11,926 (3.3)	11,743 (3.3)	183 (5.5)	<0.001
**Asthma**	44,563 (12.2)	44,081 (12.2)	482 (14.5)	<0.001
**Emphysema**	4366 (1.2)	4274 (1.2)	92 (2.8)	<0.001
**TyG**	8.67 [8.30, 9.06]	8.67 [8.30, 9.06]	8.86 [8.50, 9.26]	<0.001
**TyG-BMI**	231.46 [203.06, 265.03]	231.25 [202.91, 264.75]	254.77 [223.73, 295.24]	<0.001
**TyG-Waist**	775.49 [675.34, 878.01]	774.65 [674.72, 877.11]	860.37 [761.78, 967.85]	<0.001
**TyG-WHR**	7.54 [6.77, 8.32]	7.53 [6.76, 8.31]	8.16 [7.44, 8.89]	<0.001
**TyG-ABSI**	66.53 [61.38, 71.66]	66.50 [61.35, 71.62]	70.18 [65.52, 74.83]	<0.001
**TyG-WHtR**	4.59 [4.05, 5.16]	4.59 [4.05, 5.16]	5.07 [4.53, 5.69]	<0.001
**Blood pressure medication**	63,710 (17.5)	62,350 (17.3)	1360 (40.9)	<0.001
**Cholesterol lowering medication**	50,660 (13.9)	49,467 (13.7)	1193 (35.9)	<0.001
**Insulin**	3492 (1.0)	3385 (0.9)	107 (3.2)	0.001
**CKM stage**				
0	66,290 (18.2)	66,099 (18.3)	191 (5.7)	<0.001
1	76,318 (21.0)	75,877 (21.0)	441 (13.3)	
2	213,125 (58.6)	210,837 (58.5)	2288 (68.8)	
3	8174 (2.2)	7768 (2.2)	406 (12.2)	

BMI, body mass index; WHR, waist to hip ratio; WHtR, waist to height ratio; ABSI, a body shape index; SBP, systolic blood pressure; DBP, diastolic blood pressure; TyG, Triglyceride-Glucose Index; CKM, Cardiovascular-kidney-metabolic syndrome; AS, Aortic stenosis. Bold text denotes main variables; indented rows are subcategories.

Bold values indicate the primary characteristics. Indented items represent subcategories.

### Associations between TyG and obesity-derived indices with incident AS

**Figure 1:**
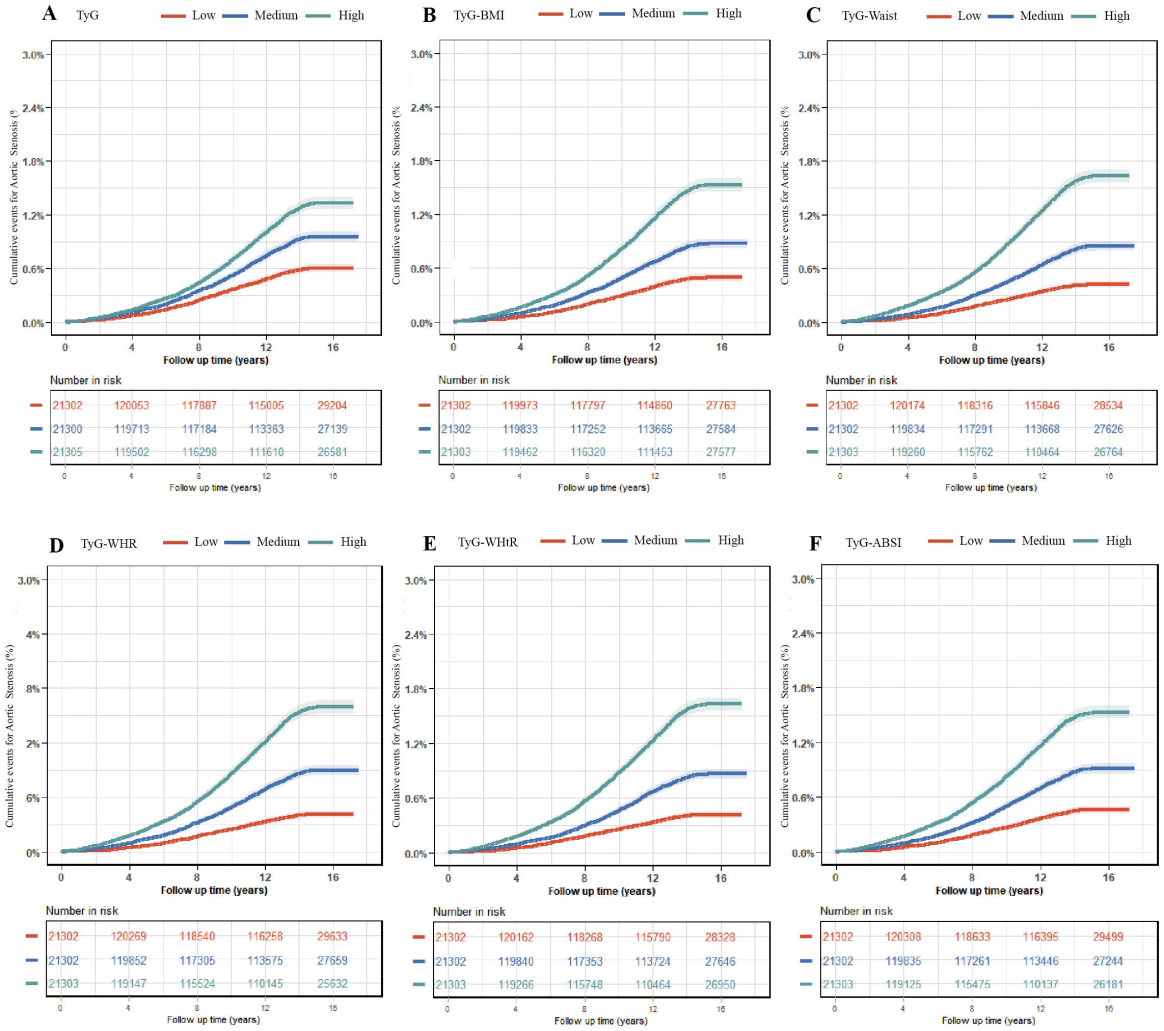
Kaplan–Meier curves of AS according to the quartiles of the TyG-related index in participants with CKM syndrome stages 0–3. **(A)** TyG index; **(B)** TyG-BMI index; **(C)** TyG-Waist index; **(D)** TyG-WHR index; **(E)** TyG-WHtR index; **(F)** TyG-ABSI index. TyG, triglyceride-glucose index; CKM, Cardiovascular–Kidney–Metabolic; AS, Aortic stenosis.

Throughout a median follow-up of 15.25 years, 3,326 individuals developed incident AS, with incidence 9 and 4 per 10,000 person-years among men and women respectively. Survival analysis demonstrated that patients in higher tertiles of TyG had significantly greater cumulative incidence of AS compared with the lowest tertile (log-rank P <0.001). Moreover, similar trends have been seen for obesity-derived indices (**Figure 1**).

**Figure 2:**
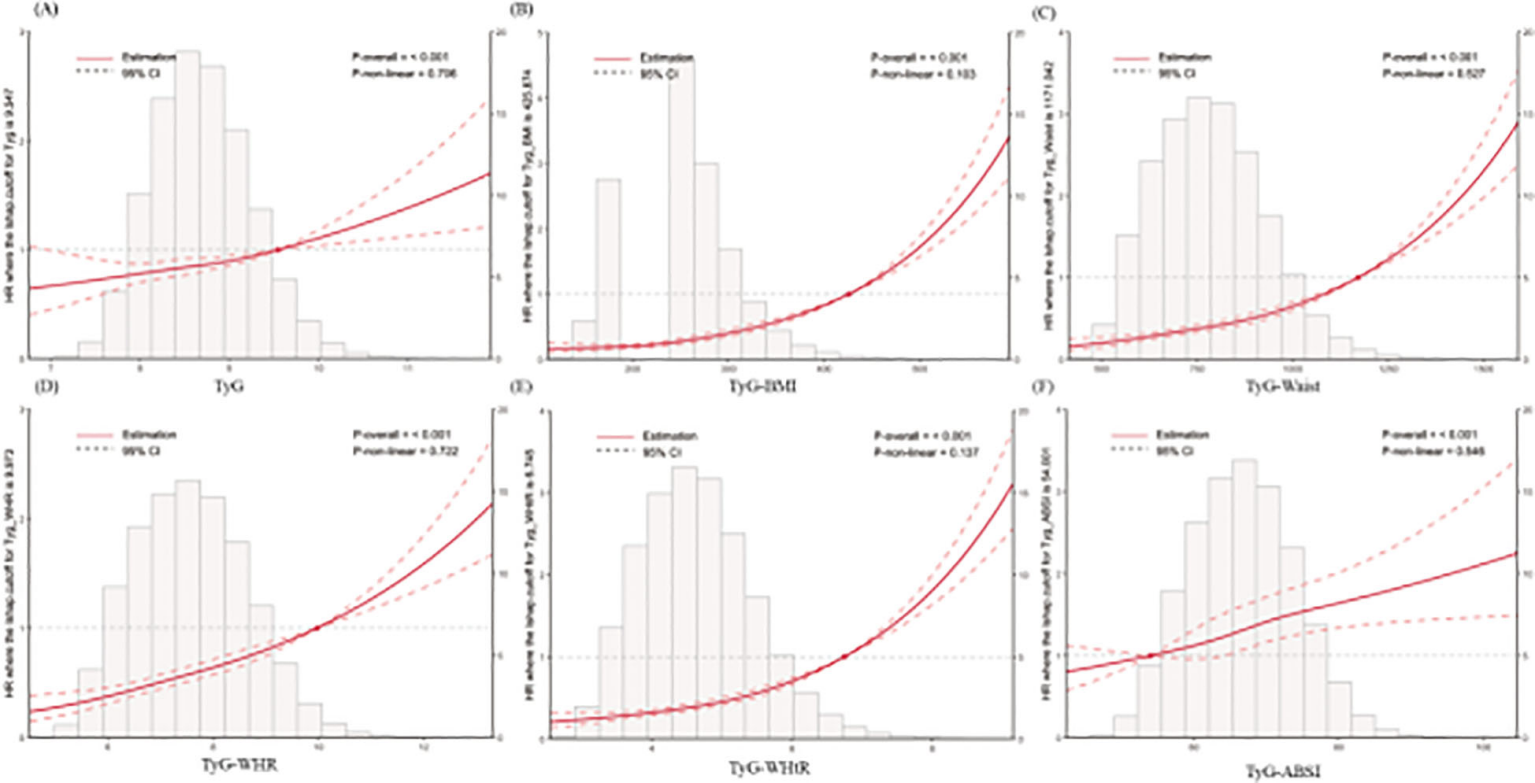
Dose-response relationship between TyG-related indicators and AS in participants with CKM syndrome stages 0–3. TyG, triglyceride-glucose index; CKM, Cardiovascular–Kidney–Metabolic; AS, Aortic stenosis.

Results from cox regression and Fine-Gray competing risk models assessing six TyG-related indices are presented in **Table 2**. Fully adjusted models showed significant positive correlation of all six indices with AS risk. In competing risk models, each SD increment in TyG was connected to 8% greater AS risk (95% CI: 4–11%). Comparable positive relationships were identified for the obesity-derived indices: TyG-BMI 24% (95% CI: 20–27%), TyG-Waist 21% (95% CI: 17–25%), TyG-WHR 14% (95% CI: 10–18%), TyG-WHtR 21% (95% CI: 17–25%), and TyG-ABSI 3% (95% CI: 0–6%). Relative to the lowest tertile, individuals in the highest tertile exhibited HRs for AS of 1.21 (95% CI: 1.11–1.32) for TyG, 1.77 (95% CI: 1.61–1.95) for TyG-BMI, 1.67 (95% CI: 1.50–1.86) for TyG-Waist, 1.44 (95% CI: 1.29–1.61) for TyG-WHR, 1.65 (95% CI: 1.49–1.83) for TyG-WHtR, and 1.13 (95% CI: 1.02–1.25) for TyG-ABSI. RCS analyses demonstrated linear dose-response relationships between all six indices and AS risk (TyG, P for non-linearity = 0.706; TyG-BMI = 0.103; TyG-Waist = 0.527; TyG-WHR = 0.722; TyG-WHtR = 0.137; TyG-ABSI = 0.546; **Figure 2**).

**Table 2:** Associations between TyG-related indices and incident AS in participants with CKM syndrome stages 0–3.

Exposures	HR (95% CI)			
	Cox proportional hazards model		Fine and gray model	
	Model 1	Model 2	Model 1	Model 2
TyG index				
Per SD increment	1.25 (1.21-1.30)	1.10 (1.06-1.14)	1.12 (1.09-1.16)	1.08 (1.04-1.11)
Tertile 1	Reference	Reference	Reference	Reference
Tertile 2	1.19 (1.08-1.31)	1.09 (1.01-1.20)	1.18 (1.08-1.29)	1.16 (1.06-1.27)
Tertile 3	1.52 (1.39-1.67)	1.19 (1.08-1.31)	1.31 (1.20-1.43)	1.21 (1.11-1.32)
TyG-BMI index				
Per SD increment	1.55 (1.50-1.60)	1.40 (1.36-1.45)	1.26 (1.23-1.29)	1.24 (1.20-1.27)
Tertile 1	Reference	Reference	Reference	Reference
Tertile 2	1.33 (1.20-1.47)	1.22 (1.10-1.35)	1.37 (1.24-1.51)	1.33 (1.21-1.47)
Tertile 3	2.42 (2.20-2.66)	1.90 (1.71-2.10)	1.88 (1.71-2.05)	1.77 (1.61-1.95)
TyG-Waist index				
Per SD increment	1.59 (1.54-1.65)	1.40 (1.35-1.45)	1.24 (1.20-1.28)	1.21 (1.17-1.25)
Tertile 1	Reference	Reference	Reference	Reference
Tertile 2	1.46 (1.31-1.63)	1.33 (1.19-1.49)	1.34 (1.20-1.49)	1.32 (1.18-1.47)
Tertile 3	2.55 (2.29-2.84)	1.95 (1.74-2.18)	1.77 (1.60-1.96)	1.67 (1.50-1.86)
TyG-WHR index				
Per SD increment	1.52 (1.46-1.58)	1.29 (1.24-1.35)	1.19 (1.15-1.23)	1.14 (1.10-1.18)
Tertile 1	Reference	Reference	Reference	Reference
Tertile 2	1.56 (1.40-1.75)	1.38 (1.23-1.54)	1.34 (1.21-1.49)	1.31 (1.18-1.46)
Tertile 3	2.37 (2.11-2.65)	1.71 (1.52-1.93)	1.57 (1.41-1.75)	1.44 (1.29-1.61)
TyG-WHtR index				
Per SD increment	1.58 (1.53-1.63)	1.40 (1.35-1.45)	1.24 (1.20-1.27)	1.21 (1.17-1.25)
Tertile 1	Reference	Reference	Reference	Reference
Tertile 2	1.45 (1.30-1.62)	1.33 (1.19-1.48)	1.37 (1.23-1.52)	1.34 (1.20-1.49)
Tertile 3	2.51 (2.27-2.78)	1.94 (1.74-2.16)	1.77 (1.60-1.95)	1.65 (1.49-1.83)
TyG-ABSI index				
Per SD increment	1.32 (1.27-1.37)	1.14 (1.10-1.19)	1.07 (1.03-1.11)	1.03 (1.00-1.06)
Tertile 1	Reference	Reference	Reference	Reference
Tertile 2	1.56 (1.40-1.75)	1.38 (1.23-1.54)	1.11 (1.00-1.22)	1.08 (1.01-1.21)
Tertile 3	2.37 (2.11-2.65)	1.71 (1.52-1.93)	1.20 (1.09-1.33)	1.13 (1.02-1.25)

Model 1 was adjusted for age, gender and race; Model 2 was further adjusted for systolic blood pressure, eGFR, Lipoprotein A, smoking status, hyperlipidemia, history of diabetes and history of emphysema. HR, hazard ratio; CI, confidence interval; SD, standard deviation; BMI, body mass index; WHR, waist to hip ratio; WHtR, waist to height ratio; ABSI, a body shape index; TyG, Triglyceride-Glucose Index; CKM, Cardiovascular-kidney-metabolic syndrome. AS, Aortic stenosis.

### Mediating role of SBP

**Figure 3:**
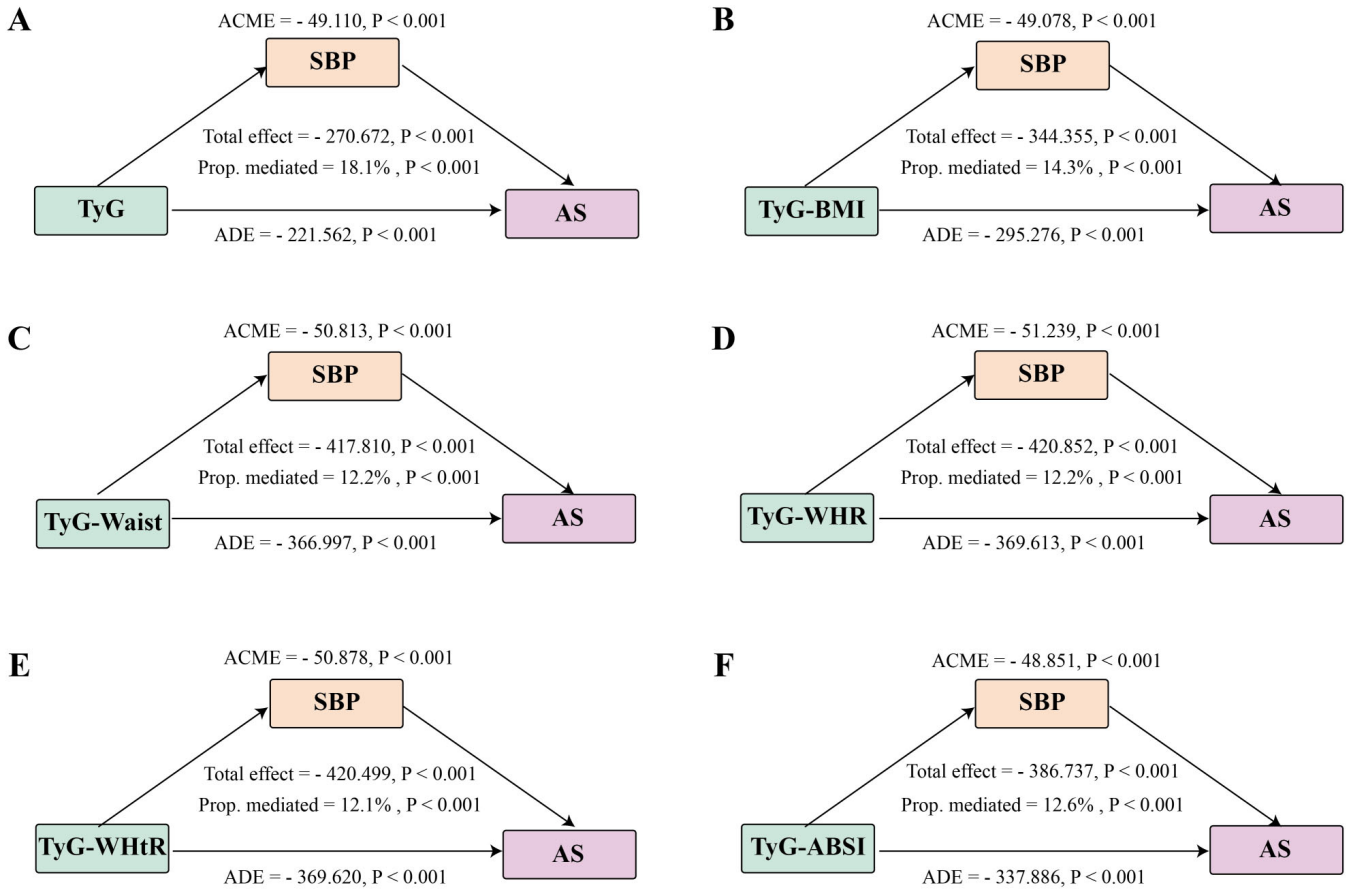
Proportion of the association between Six TyG-related indices and AS risk statistically accounted for by SBP in CKM syndrome stages 0–3. **(A)** TyG index; **(B)** TyG-BMI index; **(C)** TyG-Waist index; **(D)** TyG-WHR index; **(E)** TyG-WHtR index; **(F)** TyG-ABSI index. TyG, triglyceride-glucose index; CKM, Cardiovascular–Kidney–Metabolic; AS, Aortic stenosis; SBP, Systolic Blood Pressure.

Higher TyG and obesity-derived indices were positively associated with SBP (**Supplementary Table 4**). Each SD increase in TyG, TyG-BMI, TyG-Waist, TyG-WHR, TyG-WHtR, and TyG-ABSI corresponded to SBP increases of 2.9 (95% CI: 2.8–3.0), 4.0 (95% CI: 3.9–4.0), 4.0 (95% CI: 4.0–4.1), 4.0 (95% CI: 3.9–4.0), 2.6 (95% CI: 2.5–2.7), and 4.0 (95% CI: 4.0–4.1) mmHg, respectively. Mediation analysis suggested that SBP statistically accounted for a portion of the association between TyG-related indices and AS risk, with the estimated proportion ranging from 12.1% to 18.1% (**Figure 3**). Specifically, the statistically estimated proportion accounted for by SBP was 18.1% for TyG, and 14.3%, 12.2%, 12.2%, 12.1%, and 12.6% for TyG-BMI, TyG-Waist, TyG-WHR, TyG-WHtR, and TyG-ABSI, respectively.

### Predictive performance of TyG and obesity-derived indices for AS

Incorporating TyG-related indices into a baseline predictive model improved AS risk prediction (**Table 3**). The baseline model (adjusted for age, sex, race, SBP, eGFR, lipoprotein(a), smoking status, hyperlipidemia, diabetes, and emphysema) had a Harrell’s C-index of 0.796 (95% CI: 0.789–0.803). Models including TyG-WHtR achieved the highest C-index of 0.806 (95% CI: 0.800–0.811), while models including TyG, TyG-BMI, TyG-Waist, TyG-WHR, and TyG-ABSI had C-indices of 0.797, 0.805, 0.803, 0.800, and 0.797, respectively. Net reclassification improvement (NRI) was also maximized with TyG-WHtR (0.308; 95% CI: 0.198–0.354), whereas TyG alone yielded the lowest NRI (0.119; 95% CI: 0.085–0.169). These findings imply that TyG-WHtR most effectively enhances predictive performance for AS among CKM stages 0–3 participants.

**Table 3:** Predictive value of TyG-related indices for AS in patients with CKM syndrome stages 0–3.

Model	Harrell’s C-index (95% CI)	NRI (95% CI)
Basic model	0.796 (0.789, 0.803)	Ref
Basic model + TyG index	0.797 (0.790, 0.804)	0.119 (0.085,0.169)
Basic model + TyG-BMI index	0.805 (0.798, 0.812)	0.303 (0.238,0.354)
Basic model + TyG-Waist index	0.803 (0.797, 0.810)	0.286 (0.194,0.329)
Basic model + TyG-WHR index	0.800 (0.793, 0.806)	0.208 (0.119,0.262)
Basic model + TyG-WHtR index	0.806 (0.798, 0.811)	0.308 (0.198,0.354)
Basic model + TyG-ABSI index	0.797 (0.791, 0.804)	0.135 (0.075,0.180)

CI, confidence interval; IDI, integrated discrimination improvement index; TyG, triglyceride-glucose index; BMI, body mass index; WHtR, weight-to-height ratio; WHR, waist-to-hip ratio; AS: Aortic stenosis. The base model was adjusted for age, sex, race, systolic blood pressure, eGFR, lipoprotein(a), smoking status, hyperlipidemia, history of diabetes, and history of emphysema.

### Sensitivity and subgroup analyses

**Figure 4:**
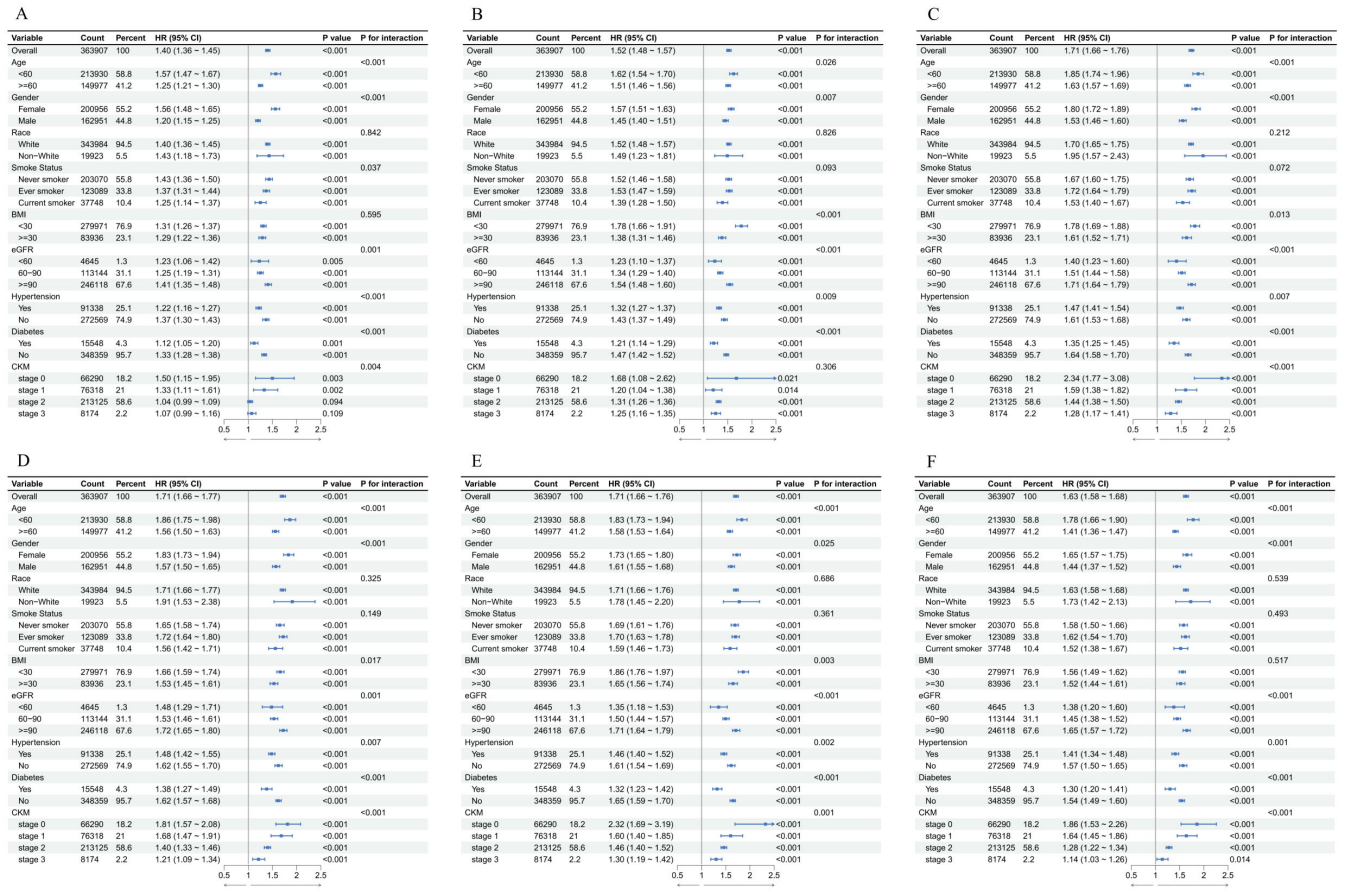
Subgroup analyses of the associations between TyG-related indices and incident AS risk in individuals with CKM syndrome stages 0–3. **(A)** TyG index; **(B)** TyG-BMI index; **(C)** TyG-Waist index; **(D)** TyG-WHR index; **(E)** TyG-WHtR index; **(F)** TyG-ABSI index. Hazard ratios (HRs) and 95% confidence intervals (CIs) were estimated per standard deviation (SD) increase in each TyG-related index. TyG, triglyceride-glucose index; BMI, body mass index; WHR, waist-to-hip ratio; WHtR, waist-to-height ratio; ABSI, a body shape index; CKM, cardiovascular-kidney-metabolic; AS, Aortic stenosis; SBP, Systolic Blood Pressure.

Subgroup analyses validated the robustness of our work (**Figure 4**). Notably, elevated TyG was connected to increased AS risk only in CKM stages 0–1, whereas obesity-derived indices maintained significant associations across CKM stages 0–3. Subgroup analyses stratified by CKM stage revealed significant effect modification for TyG, TyG-Waist, TyG-WHR, TyG-WHtR, and TyG-ABSI (P for interaction = 0.004, <0.001, <0.001, 0.001, and <0.001, respectively), whereas no significant interaction was observed for TyG-BMI (P for interaction = 0.306). Associations were consistently stronger in CKM stages 0–1 compared with stages 2–3 across all six indices, which may reflect the predominant role of insulin resistance as a metabolic driver of AS initiation in early CKM stages, whereas CKD-specific mechanisms including mineral metabolism dysregulation and uremia-associated inflammation may become increasingly dominant in advanced stages, attenuating the relative contribution of insulin resistance per se.

Furthermore, associations between 1-SD increases in TyG-related indices and AS risk were stronger in younger participants, females, those with eGFR ≥90, without hypertension or diabetes (except for TyG-Waist), and early CKM stages. No significant interactions were observed by race. Sensitivity analyses were conducted after excluding AS that occurred during the initial two years or further adjusting for medications (antihypertensives, lipid-lowering agents, and insulin), yielding consistent results (**Supplementary Table 5**, **6**).

To further address potential residual confounding effects, we conducted four additional sensitivity analyses in the respective participant subsets. In each analysis, we additionally adjusted for one variable on the basis of all covariates included in Model 2. After the additional adjustments for UACR (n=108,000), hsCRP (n=363,134), overall physical activity level (n=283,871), and dietary quality (n=342,249) respectively, the associations between the six TyG-related indices and AS risk remained significant in both the Cox and Fine-Gray models. In the Cox model, the ranges of the hazard ratios (HRs) per 1-SD increment after additional adjustment for UACR, hsCRP, physical activity, and diet were 1.11–1.45, 1.10–1.38, 1.10–1.40, and 1.11–1.41, respectively. In all four analyses, the HR was lowest for TyG and highest for the obesity-derived indices (TyG-WHtR or TyG-Waist). In the Fine-Gray model, the corresponding ranges were 1.07–1.26, 1.07–1.24, 1.08–1.26, and 1.04–1.24. In all sensitivity analyses, the risk of AS was significantly higher in the highest tertile group than in the lowest tertile group for each index (**Supplementary Tables 7**-**10**). The above results confirm that the associations between the TyG-related indices and AS risk are robust.

## Discussion

We firstly comprehensively examine correlation of six TyG-related indices and incident AS risk. In this large prospective cohort with 15.25 years of follow-up, involving 363,907 CKM stages 0–3 participants, all six indices were positively associated with AS risk. Each SD increase in TyG-related indices corresponded to an 8–24% higher risk of AS. Individuals belong to highest tertile had 1.13–1.77-fold higher risk compared to the lowest. RCS analyses indicated linear dose-response relationship, and mediation analysis indicated that SBP contributed to 12.1–18.1% of the associations. Collectively, baseline TyG-related indices, particularly TyG-WHtR, were independently associated with long-term incident AS risk and significantly improved predictive performance for AS risk.

Previous works suggested insulin resistance to be an independent cardiovascular risk factor, connected to atrial fibrillation, heart failure, and coronary heart disease (29). TyG has been recognized as a dependable biomarker for insulin resistance and is positively associated with hypertension, coronary heart disease, and stroke (29–32). Obesity further interacts with insulin resistance via chronic adipose inflammation, disrupted insulin signaling, and metabolic dysregulation (33). Combining TyG with obesity-related indices improves risk assessment for adverse cardiovascular outcomes, as demonstrated in prior studies linking TyG-WHtR and TyG-Waist to stroke and ASCVD (34).

However, few articles have examined correlations of insulin resistance indices and incident AS, with prior research primarily focused on patients with existing AS or post-valve intervention (35, 36). Our findings extend this evidence to a large CKM population, showing consistent positive correlation among six TyG-related indices and incident AS. The highest tertiles of TyG, TyG-BMI, TyG-Waist, TyG-WHR, TyG-WHtR, and TyG-ABSI were associated with 21%, 77%, 67%, 44%, 65%, and 12% higher AS risk, respectively, highlighting the combined impact of metabolic dysfunction and obesity in AS pathogenesis. Importantly, these associations were observed after a mean follow-up of 15.25 years, suggesting that baseline metabolic status, as captured by TyG-related indices, may have long-term prognostic implications for future AS incidence.

Regarding the pathophysiological mechanisms linking insulin resistance to AS, several studies have suggested that insulin resistance not only induces hyperglycemia but also triggers systemic lipid metabolic disturbances (37). Previous research has indicated that chronic inflammation and oxidative stress are key pathways activated by dysregulated lipid metabolism. Lipid metabolic disturbances can activate lipoprotein-associated phospholipase A2 (Lp-PLA2) in the valve, which catalyzes the formation of free fatty acids (FFA) and lysophosphatidylcholine (LPC) from oxidized low-density lipoprotein (oxLDL), directly promoting valvular interstitial cell calcification, upregulating osteogenic factors, and contributing to aortic cusp fibrosis, ultimately leading to AS (38). Furthermore, insulin resistance is often accompanied by increased reactive oxygen species production (39), which exacerbates oxidative stress and induces vascular endothelial dysfunction (40). Persistent lipid abnormalities and chronic inflammation may promote local valvular calcium deposition, further driving valvular stenosis (41). A prior study also demonstrated that long-term chronic insulin resistance leads to significant alterations in cardiac outcomes, particularly left ventricular hypertrophy and reduced myocardial contractility, with valvular stenosis likely playing a critical role (42). It is noteworthy that the subgroup analysis showed that the association between TyG and AS risk was statistically significant only in CKM stages 0–1, whereas no significant association was observed in CKM stages 2–3. This may be partly attributable to the possibility that, in the early stages of CKM, insulin resistance as reflected by the TyG-related indices may play a more prominent role in the initiation of AS. As CKM progresses to advanced stages, CKD-specific pathophysiological mechanisms—including mineral metabolism disorders, secondary hyperparathyroidism, vascular and valvular calcification induced by hyperphosphatemia, and uremia-related chronic inflammation—may gradually become the dominant factors in AS progression. This may in turn attenuate the relative contribution of insulin resistance itself (43, 44). In addition, the reduced variability of TyG values among patients in CKM stages 2–3 may also weaken the statistical power to detect the association in these subgroups. The above findings together suggest that the association between insulin resistance and AS may have greater clinical relevance in the early stages of CKM syndrome.

Notably, obesity, particularly central obesity, markedly elevates the likelihood of hypertension, diabetes, and other cardiovascular risk factors (14). It has been observed that obese individuals exhibit heightened sympathetic activity and increased norepinephrine release, resulting in elevated blood pressure and accelerated cardiovascular disease development (54). In our study, mediation analysis was employed to quantify the statistical proportion of the association between TyG-related indices and AS risk that was accounted for by SBP, with estimated proportions ranging from 12.1% to 18.1%. Consistently, a previous prospective study reported that SBP statistically accounted for 26.3–32.9% of a similar association, further supporting our findings (45). It is noteworthy that insulin resistance and hypertension are closely interrelated and often form a vicious cycle. Insulin resistance may contribute to elevated blood pressure through mechanisms such as activation of the sympathetic nervous system and dysregulation of the tissue renin-angiotensin-aldosterone system (RAAS). Conversely, hypertension may further exacerbate peripheral tissue insulin resistance through microvascular damage and chronic low-grade inflammation (46). Therefore, the proportion of the association statistically accounted for by SBP should not be interpreted as evidence of a confirmed causal pathway. Instead, it may reflect the shared pathophysiological interactions between metabolic dysregulation and blood pressure dysregulation in the pathways leading to AS. In addition, obesity can directly contribute to cardiovascular disease progression (11). For example, foam cells engulf cholesterol esters that deposit in the vascular wall, thickening the intima and promoting fatty streak formation (47). Persistent release of pro-inflammatory factors from visceral adipose tissue induces systemic inflammation, leading to endothelial dysfunction and further atherosclerotic progression, thereby accelerating cardiovascular disease (48).

By comparing the performance of models based on different TyG indices and their obesity-derived indices, we further assessed the predictive ability of these six TyG-related indices for AS events in CKM syndrome stages 0–3 participants. From a clinical prediction perspective, the incremental predictive values of the six indices showed substantial differences. TyG-WHtR showed the greatest improvement in predictive performance, with the C-index increasing from 0.796 to 0.806 and an NRI of 0.308, indicating that approximately 30.8% of individuals were correctly reclassified. Similarly, TyG-BMI (C-index: 0.805; NRI: 0.303) and TyG-Waist (C-index: 0.803; NRI: 0.286) also demonstrated clinically meaningful incremental predictive value. In contrast, TyG and TyG-ABSI only resulted in minor improvements in the C-index (both increased from 0.796 to 0.797), with NRI values of 0.119 and 0.135, respectively. This indicated that although these two indices had statistically significant associations with AS risk, their incremental clinical utility on the basis of traditional risk factors was limited. TyG-WHR showed a moderate degree of predictive improvement (C-index: 0.800; NRI: 0.208).

The above findings suggested that although all six TyG-related indices were independently and statistically significantly associated with AS risk, there were substantial differences in their clinical utility for risk prediction and reclassification. Indices that combine anthropometric measures of central or overall obesity with insulin resistance, including TyG-WHtR, TyG-BMI, and TyG-Waist, provided the most clinically meaningful incremental predictive value. These indices are the most suitable candidates for incorporation into AS risk stratification tools in clinical practice. WHtR is calculated from waist circumference divided by height and reflects the degree of visceral fat accumulation. This may partly explain the superior predictive performance of TyG-WHtR (49). These findings highlight the critical role of abdominal obesity combined with insulin resistance in predicting AS risk. Notably, a previous prospective study among Swedish population reported that both overall and abdominal obesity were significantly positively associated with AS, with systemic inflammation and oxidative stress probably acting as primary mediators (50). Similarly, a study in a northern Chinese population indicated that TyG-WHtR demonstrated superior predictive ability for ASCVD (34).

Collectively, baseline TyG-related indices, particularly TyG-WHtR, are independently associated with long-term incident AS risk in individuals with CKM syndrome stages 0–3. These findings highlight the potential value of incorporating readily available metabolic and anthropometric parameters into AS risk stratification strategies for individuals across the CKM spectrum. Given that TyG-related indices are derived from routine clinical measurements — fasting triglycerides, glucose, and simple anthropometric indices — their application imposes minimal additional burden on clinical workflows, rendering them practical candidates for population-level screening and early identification of high-risk individuals. They provide new insights for early detection of high-risk patients and offer a theoretical basis for precision prevention of incident AS in the early CKM population.

The study possesses multiple strengths. First, it represents the first comprehensive evaluation of the correlation of TyG indices, their obesity-derived indices, and AS risk. The large-scale, long-term prospective follow-up of over 300,000 CKM syndrome stages 0–3 participants strengthen the reliability of the findings. Second, by employing Cox regression and Fine–Gray competing risk models, we accounted for the potential confounding effect of mortality on outcomes. Third, mediation analysis confirmed the significant role of SBP in the correlation. Fourth, sensitivity analyses reinforced the robustness of the results. Finally, by constructing and comparing prediction models that incorporated the six TyG-related indices, we demonstrated that the obesity-derived indices, particularly TyG-WHtR, provided clinically meaningful incremental predictive value beyond traditional risk factors. These findings suggest that abdominal obesity and visceral fat accumulation are key determinants of AS risk.

The present study has several limitations. First, AS events were ascertained solely through inpatient records associated with ICD-10 codes. This approach may lead to underdiagnosis of asymptomatic or mild AS cases, as the valvular lesions in these patients may remain clinically silent and thus not be captured by hospitalization records. This suggests that the reported risk estimates may represent conservative approximations of the true biological effect, and that the actual association between TyG-related indices and AS risk may be stronger than the reported findings. Second, these codes do not contain information on hemodynamic severity. Since the cohort lacked systematic echocardiographic data, we were unable to stratify AS cases according to severity or to examine whether the association between TyG-related indices and AS risk differed by disease severity. Future prospective studies incorporating comprehensive echocardiographic assessments will help to clarify potential severity-specific associations. Third, TyG and its obesity-derived indices were assessed only at baseline. This limits the evaluation of their longitudinal trajectories and cumulative exposure. Consequently, we were unable to determine whether the observed associations primarily reflect baseline metabolic status or dynamic changes in metabolic health during follow-up. Although the study employed a prospective design and sensitivity analyses excluded participants who developed AS events or were lost to follow-up within the first 2 years, the possibility of reverse causation cannot be completely ruled out. Therefore, the results of this study should be interpreted as the association between baseline TyG-related indices and the long-term future risk of AS incidence, rather than as dynamic risk monitoring. Future prospective studies with repeated metabolic measurements will help further elucidate whether time-cumulative TyG exposure provides additional predictive value beyond baseline assessment. Fourth, the SBP mediation analysis relied on baseline cross-sectional measurements, which precludes establishing the temporal sequence required for causal mediation inference. Therefore, the reported proportion mediated should be interpreted as a statistical association within shared pathophysiological pathways rather than as evidence of a confirmed causal mechanism. Fifth, residual confounding could not be completely eliminated, limiting the accuracy of causal inference. Finally, several factors limit the generalizability of these findings. Participants with CKM stage 4 (Defined as those with established cardiovascular disease including heart failure, atrial fibrillation, coronary heart disease, stroke, or peripheral arterial disease) were excluded by design. Consequently, the findings are not directly applicable to this highest-risk population, and whether TyG-related indices carry similar predictive value in individuals with pre-existing cardiovascular disease remains to be investigated. Additionally, UK Biobank participants are subject to well-documented volunteer selection bias, being generally healthier, more physically active, and of higher socioeconomic status than the general UK population. Furthermore, approximately 94.5% of participants are of White ethnicity, substantially limiting racial and ethnic diversity. These factors may result in an underestimation of the true associations in higher-risk or more ethnically diverse populations, and restrict the direct applicability of the derived risk thresholds to non-European populations. Therefore, external validation across diverse racial, ethnic, and clinical populations is strongly warranted before these findings are translated into broader clinical practice.

## Data Availability

Publicly available datasets were analyzed in this study. This data can be found here: https://biobank.ndph.ox.ac.uk/showcase/.
