# Supplementary material for: Association of triglyceride–glucose and obesity-derived indices with the risk of aortic stenosis among individuals in cardiovascular–kidney–metabolic syndrome stages 0–3: a prospective cohort study from the UK Biobank
Source: Front Endocrinol (Lausanne). 2026 Jul 7;17:1844215. doi: 10.3389/fendo.2026.1844215 (PMC13385274; doi:10.3389/fendo.2026.1844215)

**Association of Triglyceride–Glucose and Obesity-Derived Indices with the Risk of Aortic Stenosis Among Individuals in Cardiovascular–Kidney–Metabolic (CKM) Syndrome Stages 0–3: A Prospective Cohort Study from the UK Biobank**

**Table S1.** The disease corresponds to ICD 10 code.

**Table S2.** Detailed definition of cardiovascular-kidney-metabolic syndrome.

**Table S3.** CKD-EPI equations for estimating GFR on the natural scale expressed for specified sex, serum creatinine or serum cystatin C.

**Table S4.** The relationship between TYG-related indicators and SBP.

**Table S5.** Sensitivity analysis for the associations between TyG-related indices and cardiovascular disease incidence in individuals with cardiovascular-kidney-metabolic syndrome stages 0-3: excluding participants who developed outcome within the first 2 years of follow-up

**Table S6.** Sensitivity analysis for the associations between TyG-related indices and cardiovascular disease incidence in individuals with cardiovascular-kidney-metabolic syndrome stages 0-3: additionally adjusting for the impact of medication use, including antihypertensive, lipid-lowering, and glucose-lowering medications.

**Table S7.** Sensitivity analysis for the associations between TyG-related indices and aortic valve stenosis incidence in individuals with cardiovascular-kidney-metabolic syndrome stages 0-3: additionally adjusting for urinary albumin-to-creatinine ratio (UACR) among participants with available UACR data.

**Table S8.** Sensitivity analysis for the associations between TyG-related indices and aortic valve stenosis incidence in individuals with cardiovascular-kidney-metabolic syndrome stages 0-3: additionally adjusting for urinary albumin-to-creatinine ratio (UACR) among participants with available CRP data.

**Table S9.** Sensitivity analysis for the associations between TyG-related indices and aortic valve stenosis incidence in individuals with cardiovascular-kidney-metabolic syndrome stages 0-3: additionally adjusting for urinary albumin-to-creatinine ratio (UACR) among participants with available physical activity data.

**Table S10.** Sensitivity analysis for the associations between TyG-related indices and aortic valve stenosis incidence in individuals with cardiovascular-kidney-metabolic syndrome stages 0-3: additionally adjusting for urinary albumin-to-creatinine ratio (UACR) among participants with available diet data.

**Figure S1.** Flow diagram showing the derivation of the final study population.

**Table S1.** The disease corresponds to ICD 10 code.

| Disease | ICD10 |
| --- | --- |
| Rheumatic valve disease | I05, I06, I07 |
| Non-rheumatic valve disease | I34, I35, I36 |
| Congenital valve disease | Q22, Q23 |
| Endocarditis with valve disease | I38, I39 |
| Pulmonary valve disease | I37 |
| Hypertension | I48 |
| Diabetes | E11 |
| Chronic renal disease | N18 |
| Aortic valve stenosis | I350, I352 |
| Emphysema | J43 |
| Asthma | J45 |
| Bronchitis | J20, J40, J41, J42 |

**Table S2.** Detailed definition of cardiovascular-kidney-metabolic syndrome

| **Stages** | **Definition** |
| --- | --- |
| Stage 0 | Stage 0 is defined as the absence of risk factors for cardiovascular-kidney-metabolic (CKM) syndrome, including normal body mass index (BMI), waist circumference (WC), blood glucose, systolic blood pressure (SBP), diastolic blood pressure (DBP), lipid levels, renal function, and no evidence of cardiovascular disease (CVD). |
| Stage 1 | Stage 1 was characterized by the existence of at least one of the following metabolic dysfunctions: (1) BMI ≥25 kg/m^2^; (2) WC ≥88/102 cm in women/men; (3) prediabetes: 5.7%≤ glycated hemoglobin (HbA1c) ≤6.4%. |
| Stage 2 | Stage 2 is defined as the presence of moderate to high-risk chronic kidney disease (CKD) and the existence of one or more of the following metabolic risk factors: (1) hypertension; (2) diabetes; (3) triglycerides (TG) ≥ 135 mg/dL; (4) diagnosis of metabolic syndrome.  The moderate to high-risk CKD was defined as an estimated Glomerular Filtration Rate (eGFR) between 30 and 60 mL/min/1.73 m^2^, and eGFR was calculated using the CKD-EPI 2021 creatinine-based equation proposed by the Chronic Kidney Epidemiology Collaboration.  Hypertension was defied as a self-reported history of hypertension, and/or SBP ≥130 mmHg, and/or DBP ≥80 mmHg, and/or the intake of anti-hypertensive medications.  Diabetes was defined as HbA1c ≥6.4%, and/or a diagnostic history of diabetes before baseline, and/or the intake of glucose-lowering medications.  Metabolic syndrome is defined as meeting at least three of the following criteria: (1) increased WC; (2) reduced high-density lipoprotein cholesterol (HDL-C < 40 mg/dL in men or < 50 mg/dL in women); (3) high TG (TG > 150 mg/dL); (4) elevated BP (SBP ≥ 130 mmHg or DBP ≥ 80 mmHg); or (5) prediabetes. |
| Stage 3 | CKM Stage 3 was defined by the presence of very-high-risk CKD per KDIGO criteria (GFR categories 4-5 or KDIGO heat map risk stratification) or a 10-year predicted CVD risk ≥20% using the AHA PREVENT equations. The PREVENT equations were applied to adults aged 30-79 years, with participants ≥80 years conservatively assigned an age of 79 years for risk estimation. Variables outside PREVENT-defined ranges (total cholesterol: 130- 320 mg/dL; HDL: 20-100 mg/dL; systolic blood pressure: 90-200 mmHg; eGFR: 14-140 mL/min/1.73 m²) were truncated to boundary values. |
| Stage 4 | CKM Stage 4 identified individuals with established CVD (coronary heart disease, stroke, atrial fibrillation, heart failure, or peripheral artery disease) documented through primary care records, hospitalization data, death registries, or self-reported diagnoses, occurring in the context of excess/dysfunctional adiposity, CKM risk factors, or CKD. |

**Table S3.** CKD-EPI equations for estimating GFR on the natural scale expressed for specified sex, serum creatinine or serum cystatin C.

| **Coeflicients** | **Sex** | **Serum Creatinine (mg/dL)** | **Serum Cystatin C (mg/L)** | **Equation** |
| --- | --- | --- | --- | --- |
| CKD-EPI creatinine-cystatin C equation | | | | |
| AS, new | Female | ≤0.7 | ≤0.8 | GFR=135×(Scr/0.7)-0.219×(Scys/0.8)-0.323×0.9961Age×0.963 |
|  |  |  | ＞0.8 | GFR=135×(Scr/0.7)-0.219×(Scys/0.8)-0.778×0.9961Age×0.963 |
|  |  | ＞0.7 | ≤0.8 | GFR=135×(Scr/0.7)-0.544×(Scys/0.8)-0.323×0.9961 Age×0.963 |
|  |  |  | ＞0.8 | GFR=135×(Scr/0.7)-0.544×(Scys/0.8)-0.778×0.9961 Age×0.963 |
|  | Male | ≤0.9 | ≤0.8 | GFR=135×(Scr/0.9)-0.144×(Scys/0.8)-0.323×0.9961 Age |
|  |  |  | ＞0.8 | GFR=135×(Scr/0.9)-0.144×(Scys/0.8)-0.778×0.9961 Age |
|  |  | ＞0.9 | ≤0.8 | GFR=135×(Scr/0.9)-0.544×(Scys/0.8)-0.323×0.9961 Age |
|  |  |  | ＞0.8 | GFR=135×(Scr/0.9)-0.544×(Scys/0.8)-0.778×0.9961 Age |

The CKD-EPI Creatinine-Cystatin C Age, Sex Equation (2021) can be expressed as a single equation 135×min(Scr/k,1)α×max(Scr/k,1)-0.544×min(Scys/0.8,1)-0.323×max(Scys/0.8,1)-0.778×0.9961 Age×0.963 [if female]where Scr is serum creatinine Scys is serum cystatin C, k is 0.7 for females and 0.9 males, α is -0.219 for females and -0.144 for males,min indicates the minimum of Scr/k or 1, max indicates the maximum of Scr/k or 1.

**Table S4.** The relationship between TYG-related indicators and SBP.

| **Metabolites (Per SD increment)** | **Estimate (95% CI)** | **P-value** |
| --- | --- | --- |
| TyG index | 2.9 (2.8, 3.0) | <0.001 |
| TyG-BMI index | 4.0 (3.9, 4.0) | <0.001 |
| TyG-Waist index | 4.0 (4.0, 4.1) | <0.001 |
| TyG-WHR index | 4.0 (3.9, 4.0) | <0.001 |
| TyG-ABSI index | 2.6 (2.5, 2.7) | <0.001 |
| TyG-WHtR index | 4.0 (4.0, 4.1) | <0.001 |

**Table S5.** Sensitivity analysis for the associations between TyG-related indices and cardiovascular disease incidence in individuals with cardiovascular-kidney-metabolic syndrome stages 0-3: excluding participants who developed outcome within the first 2 years of follow-up

| **Exposures** | **HR (95% CI)** | | | | |
| --- | --- | --- | --- | --- | --- |
|  | **Cox proportional hazards model** | | | **Fine and Gray model** | |
|  | **Model 1** | | **Model 2** | **Model 1** | **Model 2** |
| TyG index |  | | | | |
| Per SD increment | 1.26 (1.21-1.30) | 1.10 (1.06-1.14) | | 1.12 (1.09-1.16) | 1.07 (1.04-1.11) |
| Tertile 1 | Reference | Reference | | Reference | Reference |
| Tertile 2 | 1.17 (1.07-1.29) | 1.08 (1.01-1.19) | | 1.17 (1.07-1.28) | 1.16 (1.06-1.27) |
| Tertile 3 | 1.52 (1.39-1.67) | 1.19 (1.08-1.31) | | 1.30 (1.19-1.42) | 1.21 (1.11-1.32) |
| TyG-BMI index |  |  | |  |  |
| Per SD increment | 1.55 (1.50-1.59) | 1.40 (1.35-1.45) | | 1.25 (1.21-1.28) | 1.23 (1.19-1.26) |
| Tertile 1 | Reference | Reference | | Reference | Reference |
| Tertile 2 | 1.34 (1.20-1.49) | 1.23 (1.10-1.37) | | 1.35 (1.22-1.50) | 1.32 (1.20-1.46) |
| Tertile 3 | 2.43 (2.20-2.68) | 1.90 (1.72-2.11) | | 1.87 (1.71-2.05) | 1.76 (1.60-1.94) |
| TyG-Waist index |  |  | |  |  |
| Per SD increment | 1.59 (1.54-1.65) | 1.40 (1.35-1.45) | | 1.20 (1.16-1.24) | 1.16 (1.12-1.20) |
| Tertile 1 | Reference | Reference | | Reference | Reference |
| Tertile 2 | 1.45 (1.30-1.63) | 1.33 (1.19-1.49) | | 1.30 (1.16-1.45) | 1.30 (1.16-1.45) |
| Tertile 3 | 2.54 (2.27-2.83) | 1.95 (1.74-2.18) | | 1.74 (1.56-1.92) | 1.65 (1.48-1.84) |
| TyG-WHR index |  |  | |  |  |
| Per SD increment | 1.51 (1.45-1.58) | 1.29 (1.23-1.34) | | 1.16 (1.12-1.20) | 1.12 (1.08-1.15) |
| Tertile 1 | Reference | Reference | | Reference | Reference |
| Tertile 2 | 1.55 (1.38-1.73) | 1.36 (1.22-1.53) | | 1.32 (1.20-1.45) | 1.30 (1.16-1.44) |
| Tertile 3 | 2.34 (2.08-2.63) | 1.69 (1.50-1.91) | | 1.55 (1.40-1.73) | 1.42 (1.28-1.60) |
| TyG-WHtR index |  |  | |  |  |
| Per SD increment | 1.59 (1.53-1.65) | 1.40 (1.34-1.45) | | 1.22 (1.20-1.25) | 1.20 (1.15-1.25) |
| Tertile 1 | Reference | Reference | | Reference | Reference |
| Tertile 2 | 1.43 (1.28-1.60) | 1.31 (1.17-1.47) | | 1.35 (1.20-1.50) | 1.30 (1.16-1.45) |
| Tertile 3 | 2.50 (2.25-2.77) | 1.92 (1.72-2.15) | | 1.75 (1.58-1.90) | 1.62 (1.45-1.80) |
| TyG-ABSI index |  |  | |  |  |
| Per SD increment | 1.32 (1.27-1.37) | 1.14 (1.10-1.19) | | 1.05 (1.00-1.10) | 1.02 (1.00-1.04) |
| Tertile 1 | Reference | Reference | | Reference | Reference |
| Tertile 2 | 1.56 (1.40-1.75) | 1.38 (1.23-1.54) | | 1.11 (1.00-1.22) | 1.07 (1.01-1.20) |
| Tertile 3 | 2.37 (2.11-2.65) | 1.71 (1.52-1.93) | | 1.20 (1.09-1.33) | 1.11 (1.01-1.24) |

**Table S6.** Sensitivity analysis for the associations between TyG-related indices and cardiovascular disease incidence in individuals with cardiovascular-kidney-metabolic syndrome stages 0-3: additionally adjusting for the impact of medication use, including antihypertensive, lipid-lowering, and glucose-lowering medications.

| **Exposures** | **HR (95% CI)** | | | | |
| --- | --- | --- | --- | --- | --- |
|  | **Cox proportional hazards model** | | | **Fine and Gray model** | |
|  | **Model 1** | | **Model 2** | **Model 1** | **Model 2** |
| TyG index |  | | | | |
| Per SD increment | 1.26 (1.21-1.30) | 1.10 (1.06-1.14) | | 1.11 (1.09-1.16) | 1.06 (1.04-1.11) |
| Tertile 1 | Reference | Reference | | Reference | Reference |
| Tertile 2 | 1.17 (1.07-1.29) | 1.08 (1.00-1.19) | | 1.17 (1.07-1.28) | 1.15 (1.06-1.27) |
| Tertile 3 | 1.50 (1.38-1.65) | 1.18 (1.08-1.29) | | 1.32 (1.18-1.42) | 1.22 (1.11-1.32) |
| TyG-BMI index |  |  | |  |  |
| Per SD increment | 1.54 (1.50-1.58) | 1.41 (1.34-1.46) | | 1.24 (1.20-1.26) | 1.22 (1.18-1.24) |
| Tertile 1 | Reference | Reference | | Reference | Reference |
| Tertile 2 | 1.32 (1.20-1.49) | 1.23 (1.11-1.36) | | 1.35 (1.22-1.50) | 1.32 (1.20-1.45) |
| Tertile 3 | 2.42 (2.21-2.67) | 1.90 (1.70-2.10) | | 1.86 (1.72-2.06) | 1.75 (1.60-1.92) |
| TyG-Waist index |  |  | |  |  |
| Per SD increment | 1.56 (1.52-1.65) | 1.42 (1.34-1.45) | | 1.21 (1.16-1.24) | 1.14 (1.12-1.20) |
| Tertile 1 | Reference | Reference | | Reference | Reference |
| Tertile 2 | 1.43 (1.30-1.61) | 1.31 (1.18-1.46) | | 1.30 (1.16-1.45) | 1.30 (1.16-1.45) |
| Tertile 3 | 2.54 (2.27-2.82) | 1.93 (1.7-2.16) | | 1.72 (1.55-1.91) | 1.65 (1.48-1.84) |
| TyG-WHR index |  |  | |  |  |
| Per SD increment | 1.51 (1.45-1.58) | 1.29 (1.23-1.34) | | 1.12 (1.10-1.20) | 1.14 (1.08-1.15) |
| Tertile 1 | Reference | Reference | | Reference | Reference |
| Tertile 2 | 1.55 (1.38-1.73) | 1.34 (1.20-1.51) | | 1.32 (1.20-1.45) | 1.30 (1.16-1.44) |
| Tertile 3 | 2.34 (2.08-2.63) | 1.68 (1.50-1.90) | | 1.55 (1.40-1.73) | 1.42 (1.28-1.60) |
| TyG-WHtR index |  |  | |  |  |
| Per SD increment | 1.59 (1.53-1.65) | 1.40 (1.34-1.45) | | 1.22 (1.20-1.25) | 1.20 (1.15-1.25) |
| Tertile 1 | Reference | Reference | | Reference | Reference |
| Tertile 2 | 1.43 (1.28-1.60) | 1.31 (1.17-1.47) | | 1.35 (1.20-1.50) | 1.30 (1.16-1.45) |
| Tertile 3 | 2.50 (2.25-2.77) | 1.92 (1.72-2.15) | | 1.75 (1.58-1.90) | 1.62 (1.45-1.80) |
| TyG-ABSI index |  |  | |  |  |
| Per SD increment | 1.32 (1.27-1.37) | 1.12 (1.08-1.17) | | 1.03 (1.00-1.09) | 1.01 (1.00-1.04) |
| Tertile 1 | Reference | Reference | | Reference | Reference |
| Tertile 2 | 1.56 (1.40-1.75) | 1.38 (1.23-1.54) | | 1.12 (1.00-1.22) | 1.06 (1.01-1.20) |
| Tertile 3 | 2.35 (2.10-2.65) | 1.70 (1.52-1.93) | | 1.21 (1.10-1.33) | 1.10 (1.01-1.24) |

**Table S7.** Sensitivity analysis for the associations between TyG-related indices and aortic valve stenosis incidence in individuals with cardiovascular-kidney-metabolic syndrome stages 0-3: additionally adjusting for urinary albumin-to-creatinine ratio (UACR) among participants with available UACR data.

| **Exposures** | **HR (95% CI)** | |
| --- | --- | --- |
|  | **Cox proportional hazards model** | **Fine and Gray model** |
|  | **Model 2 + UACR** | **Model 2 + UACR** |
| TyG index |  |  |
| Per SD increment | 1.11 (1.06-1.18) | 1.09 (1.05-1.15) |
| Tertile 1 | Reference | Reference |
| Tertile 2 | 1.20 (1.04-1.38) | 1.30 (1.14-1.47) |
| Tertile 3 | 1.26 (1.10-1.45) | 1.31 (1.15-1.49) |
| TyG-BMI index |  |  |
| Per SD increment | 1.43 (1.36-1.50) | 1.26 (1.21-1.32) |
| Tertile 1 | Reference | Reference |
| Tertile 2 | 1.32 (1.14-1.54) | 1.43 (1.24-1.65) |
| Tertile 3 | 2.01 (1.73-2.33) | 1.87 (1.62-2.16) |
| TyG-Waist index |  |  |
| Per SD increment | 1.43 (1.35-1.52) | 1.25 (1.19-1.32) |
| Tertile 1 | Reference | Reference |
| Tertile 2 | 1.69 (1.42-2.00) | 1.67 (1.43-1.96) |
| Tertile 3 | 2.31 (1.95-2.74) | 2.01 (1.70-2.96) |
| TyG-WHR index |  |  |
| Per SD increment | 1.12 (1.10-1.20) | 1.19 (1.13-1.26) |
| Tertile 1 | Reference | Reference |
| Tertile 2 | 1.32 (1.20-1.45) | 1.51 (1.28-1.78) |
| Tertile 3 | 1.55 (1.40-1.73) | 1.86 (1.57-2.21) |
| TyG-WHtR index |  |  |
| Per SD increment | 1.45 (1.37-1.53) | 1.25 (1.19-1.31) |
| Tertile 1 | Reference | Reference |
| Tertile 2 | 1.51 (1.28-1.78) | 1.52 (1.29-1.78) |
| Tertile 3 | 2.23 (1.90-2.62) | 1.91 (1.64-2.27) |
| TyG-ABSI index |  |  |
| Per SD increment | 1.18 (1.11-1.25) | 1.07 (1.00-1.11) |
| Tertile 1 | Reference | Reference |
| Tertile 2 | 1.27 (1.08-1.49) | 1.15 (1.01-1.34) |
| Tertile 3 | 1.54 (1.31-1.81) | 1.29 (1.11-1.51) |

**Table S8.** Sensitivity analysis for the associations between TyG-related indices and aortic valve stenosis incidence in individuals with cardiovascular-kidney-metabolic syndrome stages 0-3: additionally adjusting for urinary albumin-to-creatinine ratio (UACR) among participants with available CRP data.

| **Exposures** | **HR (95% CI)** | |
| --- | --- | --- |
|  | **Cox proportional hazards model** | **Fine and Gray model** |
|  | **Model 2 + CRP** | **Model 2 + CRP** |
| TyG index |  |  |
| Per SD increment | 1.10 (1.06-1.14) | 1.07 (1.04-1.11) |
| Tertile 1 | Reference | Reference |
| Tertile 2 | 1.08 (1.05-1.19) | 1.15 (1.05-1.26) |
| Tertile 3 | 1.18 (1.08-1.30) | 1.20 (1.10-1.31) |
| TyG-BMI index |  |  |
| Per SD increment | 1.38 (1.33-1.43) | 1.24 (1.20-1.28) |
| Tertile 1 | Reference | Reference |
| Tertile 2 | 1.21 (1.09-1.35) | 1.34 (1.21-1.48) |
| Tertile 3 | 1.85 (1.67-2.05) | 1.78 (1.61-1.96) |
| TyG-Waist index |  |  |
| Per SD increment | 1.37 (1.32-1.43) | 1.21 (1.17-1.25) |
| Tertile 1 | Reference | Reference |
| Tertile 2 | 1.30 (1.16-1.45) | 1.31 (1.18-1.46) |
| Tertile 3 | 1.87 (1.67-2.10) | 1.67 (1.50-1.86) |
| TyG-WHR index |  |  |
| Per SD increment | 1.27 (1.22-1.33) | 1.14 (1.10-1.18) |
| Tertile 1 | Reference | Reference |
| Tertile 2 | 1.34 (1.20-1.54) | 1.30 (1.17-1.45) |
| Tertile 3 | 1.65 (1.46-1.86) | 1.43 (1.28-1.60) |
| TyG-WHtR index |  |  |
| Per SD increment | 1.38 (1.33-1.43) | 1.25 (1.19-1.31) |
| Tertile 1 | Reference | Reference |
| Tertile 2 | 1.30 (1.17-1.46) | 1.38 (1.20-1.48) |
| Tertile 3 | 1.86 (1.33-1.98) | 1.65 (1.48-1.83) |
| TyG-ABSI index |  |  |
| Per SD increment | 1.13 (1.09-1.18) | 1.07 (1.00-1.11) |
| Tertile 1 | Reference | Reference |
| Tertile 2 | 1.16 (1.04-1.29) | 1.08 (1.00-1.20) |
| Tertile 3 | 1.32 (1.18-1.47) | 1.12 (1.01-1.25) |

**Table S9.** Sensitivity analysis for the associations between TyG-related indices and aortic valve stenosis incidence in individuals with cardiovascular-kidney-metabolic syndrome stages 0-3: additionally adjusting for urinary albumin-to-creatinine ratio (UACR) among participants with available physical activity data.

| **Exposures** | **HR (95% CI)** | |
| --- | --- | --- |
|  | **Cox proportional hazards model** | **Fine and Gray model** |
|  | **Model 2 + physical activity** | **Model 2 + physical activity** |
| TyG index |  |  |
| Per SD increment | 1.10 (1.05-1.14) | 1.08 (1.04-1.12) |
| Tertile 1 | Reference | Reference |
| Tertile 2 | 1.04 (1.00-1.16) | 1.17 (1.07-1.29) |
| Tertile 3 | 1.15 (1.03-1.28) | 1.22 (1.11-1.33) |
| TyG-BMI index |  |  |
| Per SD increment | 1.40 (1.35-1.46) | 1.25 (1.22-1.30) |
| Tertile 1 | Reference | Reference |
| Tertile 2 | 1.20 (1.06-1.35) | 1.32 (1.22-1.47) |
| Tertile 3 | 1.89 (1.68-2.05) | 1.79 (1.62-1.97) |
| TyG-Waist index |  |  |
| Per SD increment | 1.40 (1.34-1.46) | 1.22 (1.19-1.29) |
| Tertile 1 | Reference | Reference |
| Tertile 2 | 1.28 (1.12-1.46) | 1.33 (1.20-1.48) |
| Tertile 3 | 1.90 (1.66-2.17) | 1.67 (1.50-1.86) |
| TyG-WHR index |  |  |
| Per SD increment | 1.28 (1.22-1.35) | 1.15 (1.11-1.18) |
| Tertile 1 | Reference | Reference |
| Tertile 2 | 1.34 (1.17-1.53) | 1.30 (1.17-1.45) |
| Tertile 3 | 1.70 (1.48-1.95) | 1.45 (1.29-1.61) |
| TyG-WHtR index |  |  |
| Per SD increment | 1.40 (1.34-1.46) | 1.26 (1.20-1.31) |
| Tertile 1 | Reference | Reference |
| Tertile 2 | 1.22 (1.07-1.39) | 1.39 (1.22-1.48) |
| Tertile 3 | 1.82 (1.61-2.06) | 1.67 (1.49-1.84) |
| TyG-ABSI index |  |  |
| Per SD increment | 1.13 (1.09-1.18) | 1.09 (1.02-1.11) |
| Tertile 1 | Reference | Reference |
| Tertile 2 | 1.15 (1.01-1.30) | 1.08 (1.02-1.20) |
| Tertile 3 | 1.34 (1.18-1.52) | 1.12 (1.05-1.27) |

**Table S10.** Sensitivity analysis for the associations between TyG-related indices and aortic valve stenosis incidence in individuals with cardiovascular-kidney-metabolic syndrome stages 0-3: additionally adjusting for urinary albumin-to-creatinine ratio (UACR) among participants with available diet data.

| **Exposures** | **HR (95% CI)** | |
| --- | --- | --- |
|  | **Cox proportional hazards model** | **Fine and Gray model** |
|  | **Model 2 + diet scores** | **Model 2 + diet scores** |
| TyG index |  |  |
| Per SD increment | 1.11 (1.07-1.15) | 1.08 (1.04-1.12) |
| Tertile 1 | Reference | Reference |
| Tertile 2 | 1.08 (1.00-1.19) | 1.18 (1.08-1.28) |
| Tertile 3 | 1.19 (1.08-1.31) | 1.23 (1.12-1.33) |
| TyG-BMI index |  |  |
| Per SD increment | 1.41 (1.36-1.46) | 1.24 (1.21-1.28) |
| Tertile 1 | Reference | Reference |
| Tertile 2 | 1.25 (1.12-1.39) | 1.35 (1.22-1.50) |
| Tertile 3 | 1.94 (1.75-2.16) | 1.80 (1.63-1.99) |
| TyG-Waist index |  |  |
| Per SD increment | 1.41 (1.36-1.47) | 1.22 (1.19-1.29) |
| Tertile 1 | Reference | Reference |
| Tertile 2 | 1.35 (1.20-1.52) | 1.33 (1.20-1.48) |
| Tertile 3 | 1.98 (1.76-2.23) | 1.67 (1.50-1.86) |
| TyG-WHR index |  |  |
| Per SD increment | 1.30 (1.25-1.36) | 1.15 (1.11-1.19) |
| Tertile 1 | Reference | Reference |
| Tertile 2 | 1.38 (1.23-1.55) | 1.31 (1.18-1.457) |
| Tertile 3 | 1.71 (1.51-1.93) | 1.45 (1.29-1.63) |
| TyG-WHtR index |  |  |
| Per SD increment | 1.41 (1.36-1.46) | 1.22 (1.18-1.26) |
| Tertile 1 | Reference | Reference |
| Tertile 2 | 1.33 (1.19-1.50) | 1.33 (1.19-1.49) |
| Tertile 3 | 1.95 (1.74-2.18) | 1.66 (1.49-1.85) |
| TyG-ABSI index |  |  |
| Per SD increment | 1.16 (1.11-1.20) | 1.04 (1.01-1.08) |
| Tertile 1 | Reference | Reference |
| Tertile 2 | 1.19 (1.06-1.33) | 1.09 (0.98-1.21) |
| Tertile 3 | 1.35 (1.21-1.51) | 1.14 (1.03-1.27) |

**Figure S1.** Flow diagram showing the derivation of the final study population.


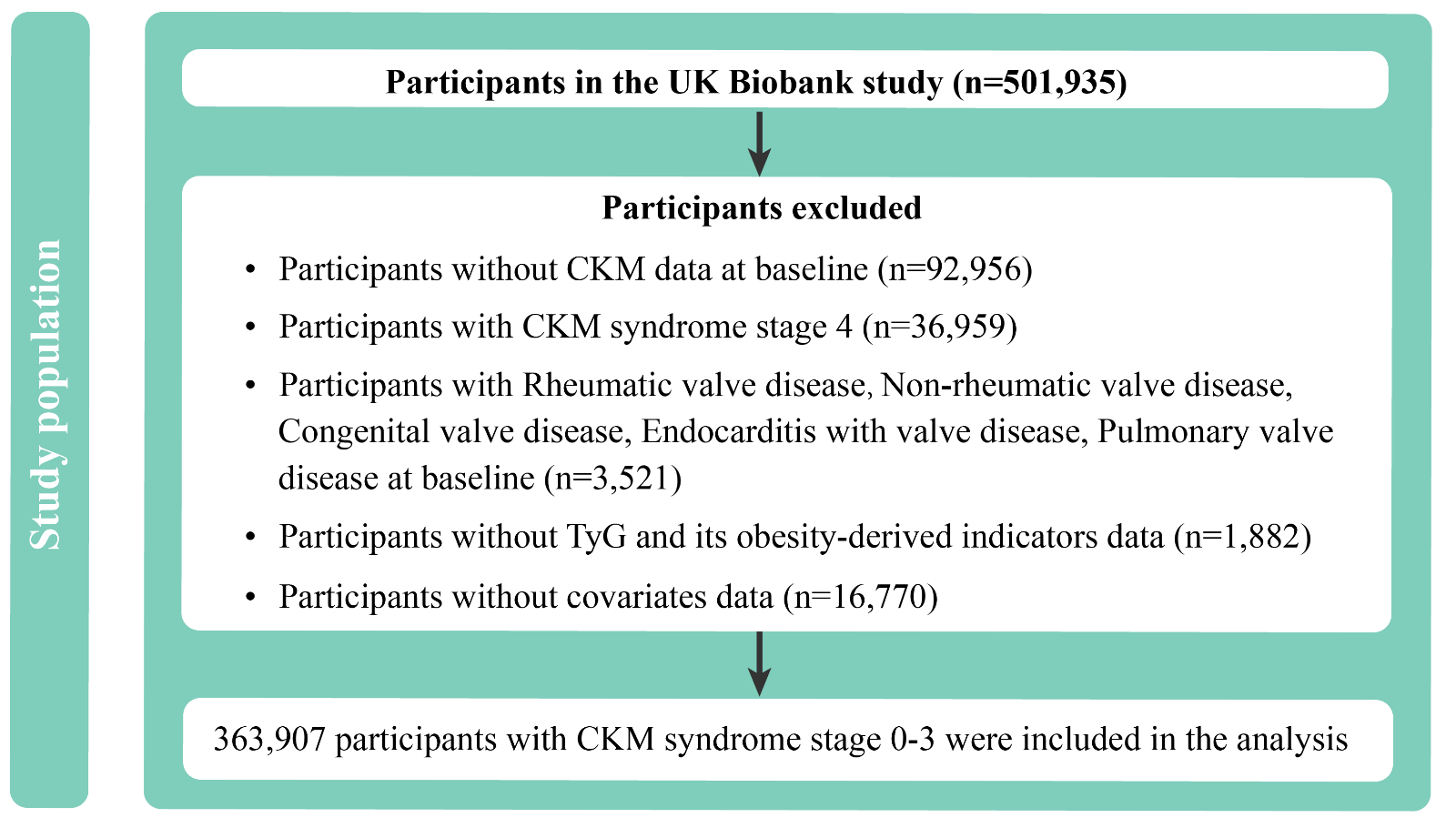

Supplement: Supplementary file 1 [file DataSheet1.docx]
